# Long-term outcome of scleral-fixated intraocular lens implantation without conjunctival peritomies and sclerotomy in ocular trauma patients

**DOI:** 10.1186/s12886-019-1172-4

**Published:** 2019-07-29

**Authors:** Han Zhao, Wanpeng Wang, Zhengping Hu, Baihua Chen

**Affiliations:** 1 Department of Ophthalmology, Second Xiangya Hospital, Central South University, Changsha, Hunan Province China; 2 Hunan Clinical Research Center of Ophthalmic Disease, Changsha, Hunan Province China; 3 Department of Ophthalmology, Xiangya Hospital, Central South University, Changsha, Hunan Province China; 4 Schepens Eye Research Institute of Massachusetts Eye and Ear, Harvard Medical School, Boston, MA USA

**Keywords:** Scleral fixated intraocular lens, Vitrectomy, Traumatic aphakic eyes, Postoperative complications

## Abstract

**Background:**

To investigate the long-term outcomes and complications of scleral-fixated intraocular lens (SFIOL) implantation without conjunctival peritomies and sclerotomy in patients with a history of ocular trauma with inadequate capsular support during primary pars plana vitrectomy or silicone oil removal.

**Methods:**

Records of ocular trauma patients who underwent implantation of SFIOL without conjunctival peritomies and sclerotomy during primary pars plana vitrectomy or silicone oil removal.

**Results:**

Sixty-nine eyes of 69 patients were included in this study. The median follow-up period was 34 months (range, 6–99 months). The average patient age at the time of surgery was 44 years old (range, 4–80 years). At the end of follow-up, the preoperative mean of best corrected visual acuity (BCVA) was 0.79 ± 0.86 log of the minimum angle of resolution (logMAR), which improved 0.20 ± 0.26 logMAR postoperatively (*P* = 0.01). BCVA improved or remained unchanged in 64 eyes (92.8%), VA decreased two lines in five eyes (7.2%). Early postoperative complications included transient corneal edema in seven eyes (10.1%), minor vitreous hemorrhage in four eyes (5.8%), transient elevated intraocular pressure (IOP) in one eye (1.4%), and hypotony in three eyes (4.3%). Late postoperative complications included persistent elevated IOP in five eyes (7.2%), epiretinal membrane formation in three eyes (4.3%), and cystoid macular edema noted in one eye (1.4%).

**Conclusions:**

Use of a scleral-fixated intraocular lens implantation without conjunctival peritomies and sclerotomy in ocular trauma patients during either primary pars plana vitrectomy or second silicone oil removal is a valuable approach for the management of traumatic aphakia in the absence of capsular support.

**Electronic supplementary material:**

The online version of this article (10.1186/s12886-019-1172-4) contains supplementary material, which is available to authorized users.

## Background

Ocular trauma is one of the main causes of severe visual impairment. An estimated 18 million people worldwide suffer from ocular trauma each year [1]. Traumatic cataracts and lens dislocations or loss are the most common and significant sequela of ocular trauma [2]. For eyes with post-traumatic cataracts or abnormal lens positions, lens removal surgery should be performed. In most cases, a traumatic cataract coincides with injury to the cornea, iris, ciliary body, retina, or sclera. Preoperative zonular or posterior capsular tears are also common in ocular trauma patients. Therefore, management of ocular trauma patients with insufficient posterior capsular support or lens dislocation is complicated by multiple factors.

However, ocular trauma patients often have deficient capsular support and preoperative zonular tear. Scleral fixated posterior chamber intraocular lens (SFIOL), anterior chamber intraocular lenses (ACIOL), iris-fixed IOL, are alternative options to intraocular lens (IOL) implantation in eyes with inadequate capsular or zonular support. This can be performed as either a primary or secondary procedure [3].

The ACIOL or iris-fixed IOL implantation can lead to a variety of complications, including corneal endothelial cell decompensation, cystoid macular edema, and glaucoma escalation, and iris chafing [4]. SFIOL implantation therefore has some relative benefits. It reduces the risk of corneal decompensation, peripheral anterior synechia, and secondary glaucoma by positioning the lens further away from anterior segment structures [5, 6]. However, when using SFIOL implantation, suture erosion and breakage, exposure of suture knot, conjunctival and sclerotomy, scleral incision and so on are all concerned [7, 8].

In order to reduce suture-related complications, some studies embedded the haptics of three-piece IOL into the scleral tunnel, but there are still some risks of postoperative hypotony, IOL slippage and lens deviation, Scleral tunnel rupture, insufficient haptic fixation power, and haptics distortion after surgery [9, 10]. Recently, Yamane et al. has modified this method and propose a new named flanged IOL fixation technology which will greatly reduce the shortcomings of previous scleral tunnel technology [11]. But there are still some things to be noticed. For example, 1) It lacks long-term data. The longest follow-up time was 36 months. The complications of slippage of haptics, IOL dislocations, and so on, still needs further observation. 2) Someone may worry about the presence of needles within the eye. if both haptic ends are not cauterized to equal amounts, it could lead to decentration. 3) The shape of the flange depends on the IOL type and the forceps to end-of-haptic distance. 4) Insertion of the trailing haptic is the most challenging step of the procedure. 5) Sometimes, the IOL haptics were implanted in the lumen of the needles with moderate difficulty, causing slight distortion or breakage of the temporal haptic. 6) there is an IOL (VA70AD; HOYA, Tokyo, Japan) with a haptic that does not form a flange on heating. 7) It needs a special 30-gauge thin-wall needle which can be obtained only in some countries [12–14].

We have developed a new surgical procedure to reduce SFIOL-related complications in ocular trauma patients without sufficient capsule support during primary pars plana vitrectomy (PPV) or secondary silicone oil removal. It uses double-loop suture to reduce the risk of suture breakage, intrascleral S-shaped suture to avoid the shortcomings of suture knots, and does not need to incise conjunctiva and sclera which minimize injury to anatomical structures of the post-traumatic eye and shorten the operative time. In this article, we report this technique and its long-term results.

## Methods

This retrospective study collected data from patients in the Department of Ophthalmology, Second Xiangya Hospital, Central South University, between January, 2010 and March, 2018. The inclusion criteria were (1) primary PPV plus lensectomy and SFIOL implantation in ocular trauma patients with dislocation or loss of normal lens or IOL accompanied by vitreous hemorrhage. (2) Primary PPV plus lensectomy and silicone oil tamponade in ocular trauma patients with dislocation or loss of normal lens or IOL accompanied by retinal detachment or intraocular foreign bodies with or without vitreous hemorrhage. Three months later, silicone oil removal surgery with secondary SFIOL implantation were carried out. (3) The degree of lens dislocation is total dislocation or beyond 210 degrees. The patients were selected for SFIOL surgery if their best corrected visual acuity (BCVA) improved. All surgeries were performed by a single surgeon baihua chen.

All patient data were collected from medical records. The refractive power of the IOL was calculated using the SRK/T formula for axial lengths between 21 and 26 mm and the Haigis formula for axial lengths exceeding this interval. The axial length was measured by A-Scan ultrasound biometry (Humphrey AB scan system, Humphrey, USA) in eyes without silicone oil or partial coherence interferometer (IOL Master, Carl Zeiss AG, Oberkochen, Germany) in eyes with silicone oil tamponade. The keratometry was determined by Pentacam (Oculus Optikgeräte GmbH, Wetzlar, Germany). If Pentacam is unable to detect keratometry due to severe corneal scar, we calculate the IOL degree by referring to the keratometric power of the opposite normal eye and refractive state of the eye before injury.

### Surgical procedure

**Fig. 1:**
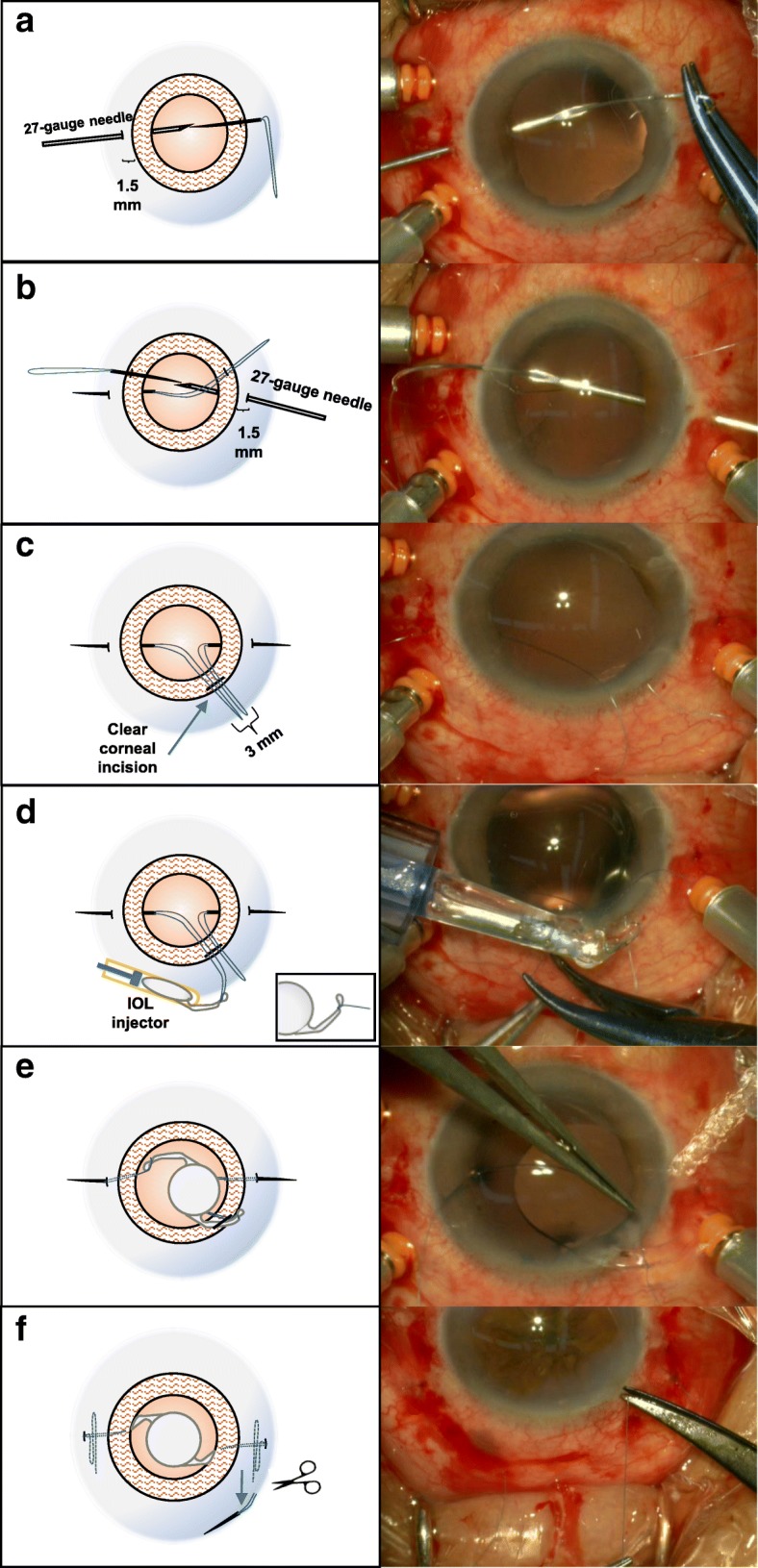
The minimally invasion knotless technique. **a**-**b**: A needle with polypropylene loop suture inserting into the lumen of the 27-gauge needle. **c** Preparation of 3-mm clear corneal incision. Polypropylene loop sutures were pulled out of the corneal tunnel incision. **d**-**e** The suture tied the IOL haptic and the foldable IOL was kept in the injector. **f** The needle is reintroduced through the existing scleral puncture site for a transscleral suture fixation and repeated three times resulting in a S-shaped pattern with suture under the sclera

The SFIOL was implanted during primary pars plana vitrectomy or secondary silicone oil removal. A needle with a 10–0 polypropylene loop suture was passed through the peripheral cornea and pulled through using a 27-gauge guide needle inserted into the posterior chamber at either the 3 o’clock or 9 o’clock axis, 1.5 mm from the limbus without conjunctival peritomies and sclerotomy (Fig. 1a, b). A 3.0 mm clear corneal tunnel incision was made in the superior temporal or nasal corneal limbus using a 3.0 mm blade to introduce the foldable IOL injector. Polypropylene loop sutures were pulled from the corneal tunnel incision (Fig. 1c). A one-piece monofocal foldable IOL (Softec HD, Lenstec, Inc.) was injected into the anterior chamber. The foldable IOL remained within the injector while the first IOL haptic was sutured in place (Fig. 1d). In order to prevent the slippage of the suture and the inclination of the IOL, the suture is tied to the outside of the maximum radian in the middle of the IOL haptic on both sides, and a small groove is formed. The IOL was then injected into the posterior chamber and the other haptic was left outside the corneal incision. The second loop suture was successively tied around the other haptic (Fig. 1e). The suture was tightened and the IOL position was adjusted in the posterior chamber. Afterward, the needle was passed through the existing scleral puncture site for intrascleral suture fixation and passing back and forth three times leaving an S-shaped pattern fixed in the sclera. The entrance of the second stitch is the exit of the first stitch, which ensures that the suture is completely located within the scleral interlayer and under the conjunctiva and sclera tissue. Last, the suture was cut without a knot (Fig. 1f). The clear corneal incision was closed with a watertight seal (Also see Video, Additional file 1).

All patients were prescribed topical 1% prednisolone acetate (Allergan Pharmaceuticals, Ireland) and 0.5% levofloxacin (Santen, Japan) four times per day, and 0.5% tropicamide phenylephrine (Santen, Japan) three times per day for 4 weeks after surgery, tapered and stopped at 6 weeks.

Postoperative data included BCVA, length of the follow-up period, the position of the IOL, corneal endothelial cell density, fundus evaluation, optical coherence tomography imaging (Heidelberg Engineering, Heidelberg, Germany) of the macula, refractive status, and IOP at each follow-up. Astigmatism caused by the IOL was measured by a vectorial method [7].

### SFIOL tilt and Decentration measurement

**Fig. 2:**
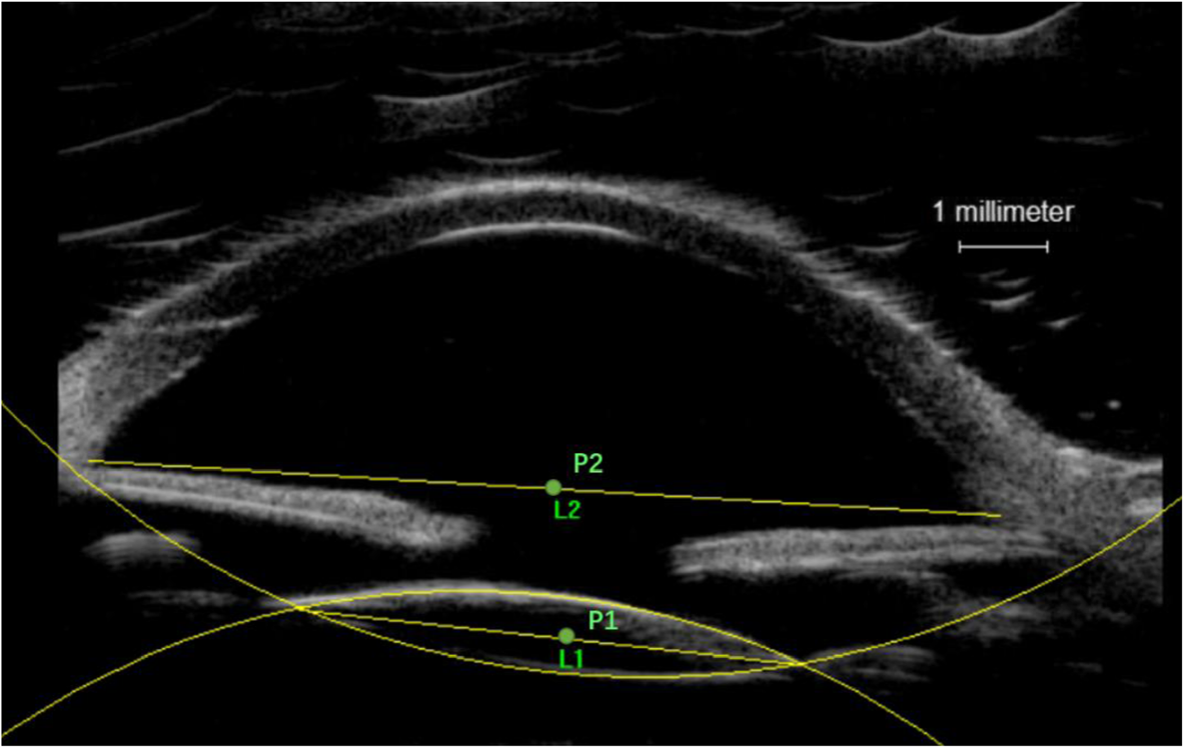
Measurement of SFIOL tilt and decentration on ultrasound biomicroscopy image using Image pro plus. P1, P2: The midpoint of the line

Postoperative SFIOL tilt and decentration were performed without pupillary dilation using ultrasound biomicroscopy (VisualSonics, Toronto, Ontario, Canada). Image pro plus 6.0 (Media Cybernetics Inc., Rockville, MD, USA) was used to measure SFIOL tilt and decentration on ultrasound images (Fig. 2). Briefly, both horizontal and vertical images were used to calculate mean SFIOL tilt and decentration. The line between the anterior chamber angles was used as the reference line (L2). Two circles were then drawn to fit the anterior and posterior arc of SFIOL. The horizontal SFIOL line passed through the circle intersections (L1). SFIOL tilt was defined as the angle between the reference line and the SFIOL line. SFIOL decentration was defined as the horizontal distance between the midpoint of L1 and L2.

### Statistical analyses

Statistical analyses were performed using SPSS (Version 22.0; IBM Corporation, Armonk, NY, USA) with statistical significance set at *P* ≤ 0.05. The BCVA is reported as mean ± SD and changes in BCVA were calculated using paired t-tests. The Wilcoxon signed-rank test was used to determine any significant differences between preoperative and postoperative BCVA or corneal endothelial cell density.

## Results

The characteristics of the study population are shown in Table 1. Sixty-nine eyes of 69 patients are included. Patients were followed up for a minimum of 6 months and a maximum of 99 months, with an average of 34 months. Of the 17(24.6%) female and 52 (75.4%) male patients, the mean age at the time of surgery was 44 years (range 4–80 years). Consistent with our previous study, the male population had a higher rate of traumatic injuries [15]. The operative eye was the right eye in 33 cases (47.8%) and the left eye in 36 cases (52.2%). Of these, 62 cases (89.8%) were traumatic lens dislocation and 7 cases (10.1%) were IOL dislocation. Forty cases (58.0%) with traumatic lens dislocation and retinal detachment received primary pars plana vitrectomy and silicone oil tamponade and were subsequently implanted with a SFIOL. A lensectomy and pars plana vitrectomy with primary SFIOL implantation was performed in 29 eyes (42.0%), whereas silicone oil removal with secondary SFIOL implantation was performed in 40 eyes (58.0%). Thirthy-one cases (45.0%) were Open-globe injury and 38 cases (55.0%) were closed-globe injury. Corneal penetration by a knife or scissors was the most common cause of open-globe injury. Before the injury, there was high myopia in three eyes, pre-operative concomitant strabismus in one eye, and a primary angle-closure glaucoma which underwent trabeculectomy in one eye.

**Table 1:** Patient characteristics

Parameters	value
No. of eyes (patients)	69 (69)
Eyes, n (%)	
Right	33 (47.8%)
Left	36 (52.2%)
Gender, n (%)	
Female	17 (24.6%)
Male	52 (75.4%)
Age	
Mean ± SD	44 ± 20
Range	4–80
Preexist lens conditions, n (%)	
Traumatic lens dislocation with retinal detachment	40 (58.0%)
Traumatic lens dislocation with vitreous hemorrhage	22 (31.9%)
Traumatic dislocation of IOLs with vitreous hemorrhage	7 (10.1%)
Type of ocular trauma, n (%)	
Open-globe injury	31 (45.0%)
Closed-globe injury	38 (55.0%)
Follow up, months	
Mean ± SD	34 ± 23
Range	6–99
Surgical procedures, n (%)	
Primary implantation	29 (42.0%)
Secondary implantation	40 (58.0%)
Ocular comorbidity, n	
High myopia	3
Concomitant strabismus	1
Glaucoma	1
IOL intraocular lens	

Table 2 shows the preoperative and postoperative visual outcomes. The mean preoperative BCVA was 0.79 ± 0.86 logMAR, which improved 0.20 ± 0.26 logMAR postoperatively (*P* = 0.01). The BCVA in 62 eyes (92.8%) improved or remained unchanged (loss of ≤1 line), and five eyes (7.2%) had a two-line decrease. The clinical characteristics of these five eyes are shown in Table 3. There was a statistically significant difference in BCVA between patients with corneal scars and those without (mean BCVA 0.08 and 0.20, *P* = 0.025). Patients with primary SFIOL implantation also had better visual outcomes than those with secondary SFIOL implantation (*P* = 0.037). The mean postoperative BCVA was comparable between open-globe injury and closed-globe injury patients. These mean BCVAs were 0.25 and 0.23, respectively (*P* = 0.106). The mean postoperative corneal endothelial cell density decreased from 2374 cells/mm^2^ to 1999 cells/mm^2^ (*P* < 0.01), and the rate of mean endothelial cell loss was 15% ± 8% at 12 months. The mean preoperative spherical equivalent (SE) was 5.16 D (range − 21.75 D to 13.87 D), which was 1.61 D (range -2D to 6 D, *P* < 0.05) post-operatively. The mean preoperative axial length was 23.47 mm (range, 16.00–27.48 mm). The mean refractive power of SFIOL was 19.47 D (range 10 D to 30 D). For total astigmatism, the mean value was 1.43 ± 2.93 D pre-operation and 0.21 ± 1.85 D post-operation. The mean SFIOL-induced astigmatism was 2.19 ± 1.50 D.

**Table 2:** Visual acuity pre- and postoperatively

Parameters	Pre-	Post-	*P* value
Spherical Equivalent refraction (D)	5.16 ± 7.01	1.61 ± 1.60	0.13
BCVA (logMAR)			
Mean ± SD	0.79 ± 0.86	0.20 ± 0.26	0.01
0.3 or better, n (%)	29 (42.0%)	61 (88.4%)	
0.4 to 1.0, n (%)	24 (34.8%)	6 (8.7%)	
1.0 or worse, n (%)	16 (23.2%)	2 (2.9%)	
Change in BCVA, n (%)			
Gained 2 or more lines	–	52 (79.7%)	
Within ±1 line of preop value	–	12 (13.1%)	
Lost 2 or more lines	–	5 (7.2%)	

BCVA best corrected visual acuity.

**Table 3:** Clinical characteristics of the five eyes with the postoperative visual decrease

Age	Preexist lens conditions	Type of ocular trauma	Preoperative BCVA (logMAR)	Postoperative BCVA (logMAR)	Surgical procedures	Ocular comorbidity	Postoperative complications
60	Traumatic lens dislocation	Penetration	0.40	0.70	Secondary implantation	Nil	ERM
66	Traumatic lens dislocation	Penetration	0.40	0.70	Secondary implantation	Nil	Higher IOL-induced astigmatism
54	Traumatic lens dislocation	Penetration	0.70	0.82	Secondary implantation	Nil	ERM
42	Traumatic lens dislocation	Penetration	0.00	0.22	Secondary implantation	Nil	Persistent elevated IOP
72	Traumatic lens dislocation	Contusion	0.10	0.22	Secondary implantation	Nil	ERM

ERM Epiretinal membrane formation, BCVA Best corrected visual acuity, IOP Intraocular pressure.

**Fig. 3:**
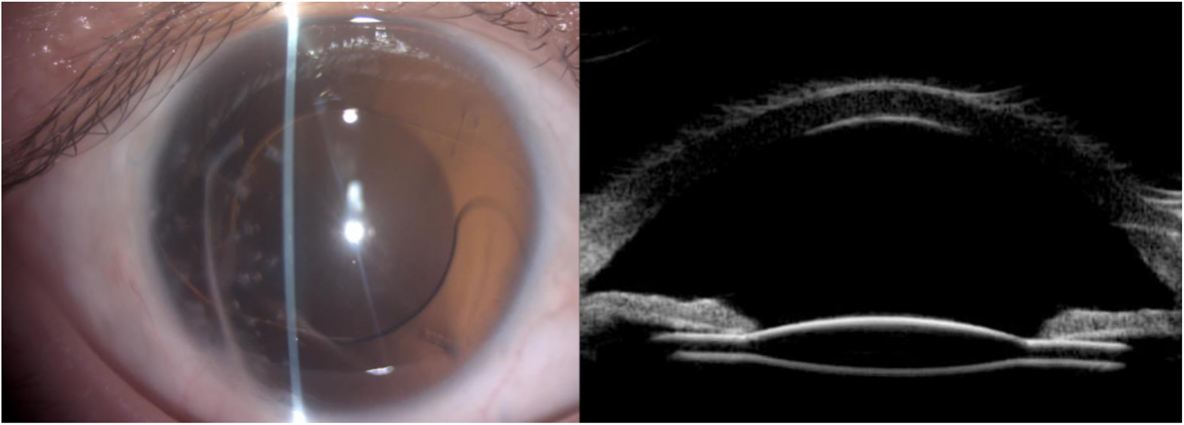
Postoperative findings at 1 year. Slit-lamp microscopy image of the IOL position. (Left) Ultrasound biomicroscopy image of a well-centered IOL. (Right)

Horizontal tilt and decentration were 2.51 ± 1.42° and 0.43 ± 0.29 mm, respectively. Vertical tilt and decentration were 2.33 ± 2.10° and 0.39 ± 0.45 mm, respectively. There were no statistically significant differences between the open-globe injury group and the closed-globe injury group, the primary and secondary groups in SFIOL tilt and decentration. Correlation assessments revealed no relationship between tilt and BCVA (*P* = 0.205), decentration and BCVA (*P* = 0.569). Figure 3 shows a typical postoperative slit-lamp microscopy image, as well as an ultrasound biomicroscopy image of a well-centered SFIOL, 1 year after surgery.

Postoperative complications are summarized in Table 4. The most common early postoperative complication was transient corneal edema (7 eyes, 10.1%) within the first 3 days after surgery. No patient suffered from visual loss or required secondary surgery. Minor vitreous hemorrhage occurred in four eyes (5.8%) because a 27-gauge needle was used to penetrate the sclera. Vitreous hemorrhage was noted within the first week after surgery and resolved without treatment. Transiently elevated IOP (> 25 mmHg) affected one eye (1.4%) within the first postoperative month and was controlled with antiglaucomatous drops. The transiently elevated IOP did not affect the final visual outcome. Hypotony was observed in three eyes (4.3%) in the first postoperative week and resolved without treatment. Persistently elevated IOP was observed in five eyes (7.2%) after 1 yr and was treated with antiglaucomatous agents which include a case of preoperative glaucoma. No patients required glaucoma surgery. Epiretinal membrane formation (ERM) was found in three eyes (4.3%). Cystoid macular edema was noted in one eye (1.4%), which was treated with anti-VEGF drugs 6 months after surgery. It resolved completely during the study period. No other complications, such as endophthalmitis or conjunctival suture erosion and breakage, were observed during the follow-up period.

**Table 4:** Postoperative complications

Complications	Patients, n (%)
Early complications (within 1 month)	
Transient corneal edema	7 (10.1%)
Minor vitreous hemorrhage	4 (6.6%)
Transient elevated intraocular pressure	1 (1.4%)
Hypotony	3 (4.3%)
Late complications (after 1 month)	
Persistent elevated intraocular pressure	5 (7.2%)
Epiretinal membrane formation	3 (4.3%)
Cystoid macular edema	1 (1.4%)

## Discussion

Traumatic cataracts and lens dislocation are the major causes of severe visual impairment after ocular trauma. In the setting of inadequate capsular support or capsular defects, SFIOL implantation is advantageous over other IOL implantation techniques. Furthermore, implantation of an ACIOL is not always possible due to defects in the iris and lack of vitreous support after pars plana vitrectomy in traumatic eyes.

At present, SFIOL is mainly divided into sutureless scleral tunnel fixation and suture fixation [16]. The sutureless intrascleral SFIOL technique was designed to eliminate suture-related complications. But there are some IOL haptic-related complications such as haptic displacement (4.1%), haptic tip extrusion (0.8%), and subconjunctival haptic (0.4%) [17]. Although Yamane recently proposed flanged haptic IOL fixation technology which can significantly reduce the haptic-related complications, more evidences and long-term observations are needed for the technical difficulty, IOL dislocation, decentration and tilt, haptic-related scleral erosion and so on. On the other hand, people’s concern about the technique of suture fixation mainly lies in suture breakage and knot-related complications [4, 18, 19]. In this study, we demonstrate a new approach to reduce the risk of suture breakage, eliminate knot-related complications, and reduce damage to eyeball structure at the same time.

First, we did not incise conjunctiva and sclera which minimizes surgical manipulations and trauma to the ocular surface and sclera. Conjunctiva and sclera preservation are particularly desirable in those population who may require surgery to control traumatic glaucoma, and helps to reduce postoperative dry eye discomfort. Second, clear corneal incision reduces the occurrence of surgically-induced corneal astigmatism [20]. Third and most importantly, the transscleral suture passing through the sclera can be rapidly fixed without any knots and thus precludes knot-related complications.

An important concern about the SFIOL is that suture knot erosion that may increase the risk of endophthalmitis. Imbedding the suture knot under the scleral flap is generally recommended. However, some papers still report the postoperative erosion rate of suture knot using this imbedding technique is 3%~ 73% [8, 21]. Although the knot-related complication rate differs from one study to another, data suggests that scleral flaps do not prevent knot erosion over the long-term [19]. We passed forth and back three times to produce S-shaped intrascleral suture to fix IOL without knotting and reduce knot-related complication. No suture erosion happened in any of our patients with maximal 99 months follow-up.

Suture fixated IOL dislocation is often due to suture breakage or slippage. The incidence of suture breakage differs between studies from 0.5 to 26.2% after mean 6 yrs follow-up [18, 22]. Longer follow-up was significantly associated with suture breakage [4]. The safety and stability of suture fixation were associated with several factors, including the fixation technique, knot-tying technique, and suture type [18, 23]. The 10–0 polypropylene suture demonstrated long-term stability for SFIOL implantation for 24.75 years [18]. Here, we used double loop 10–0 polypropylene sutures to further increase its stability and reduce breakage risk. We did not find suture breakage and spontaneous dislocation of IOL up to 99 months in our study, but long-term follow-up is still needed to determine their lifetime safety profile. Although it has been reported that increasing the diameter of sutures, such as 9–0 sutures, can reduce the rate of suture breakage, but the incidence of suture breakage (2.7% breakage with a mean follow-up of 23 months) is still higher than that of our study (0% breakage with a mean follow-up of 34 months) [24].

Tilt and decentration are important predictors of accurate IOL positioning. Unlike intracapsular IOL fixation, the IOL was fixed with suture techniques, which may increase the likelihood of IOL tilt and decentration. In our study, horizontal tilt (2.51 ± 1.42°) and decentration (0.43 ± 0.29 mm), vertical tilt (2.33 ± 2.10°) and decentration (0.39 ± 0.45 mm) are similar to other reports of scleral-sutured IOL or in-the-bag IOL [25]. In addition, there were no statistically significant differences between the open-globe and closed-globe injury groups in IOL tilt and decentration. Holladay et al. reported that spherical aberration resulting from anomalous IOL positioning was sufficient to decrease visual acuity when the decentration was more than 0.4 mm and the tilt was more than 7° [26]. The maximal SFIOL tilt and decentration in our patients was 4.41° and 1.20 mm, suggesting that the impact on the optic system is acceptably minor.

Postoperative complications with SFIOL such as retinal detachment, suprachoroidal hemorrhage, corneal edema, and persistently elevated IOP have been reported [4, 8, 27–29]. The most common postoperative complication in this series was transient corneal edema, which occurred in seven eyes (10.1%) similar to a previous study [30]. Additionally, corneal edema resolved within a week and no cases of corneal endothelial decompensation and postoperative retinal detachment were found during the follow-up period. Five eyes (7.2%) in our study developed persistently elevated IOP and were controlled with antiglaucomatous agents postoperatively. Four eyes had one or more quadrants of traumatic coloboma of the iris and angle recession. One eye is preoperative glaucoma. None of these postoperative complications resulted in a significant worsening of final visual acuity.

Multiple mechanisms may be involved in corneal endothelial cell loss, such as poor IOL positioning, surgical trauma, systemic diseases, fluid turbulence during irrigation, pars plana vitrectomy and silicone oil tamponade [31]. We recognize that endothelial cell counts begin to stabilize about 1 yr after surgery. The mean postoperative corneal endothelial cell density decreased from 2374 cells/mm2 to 1999 cells/mm2 (*P* < 0.01) and the rate of mean endothelial cell loss was 15% ± 8% at 12 months which is similar to that after phacoemulsification with IOL implantation (19.2%) in patients underwent pars plana vitrectomy with temporary silicone oil tamponade [32]. Pre-placed intraocular perfusion and the small corneal incision helps to maintain IOP stability and reduce complications such as choroidal hemorrhage, shallow anterior chamber, and hypotony maculopathy.

**Fig. 4:**
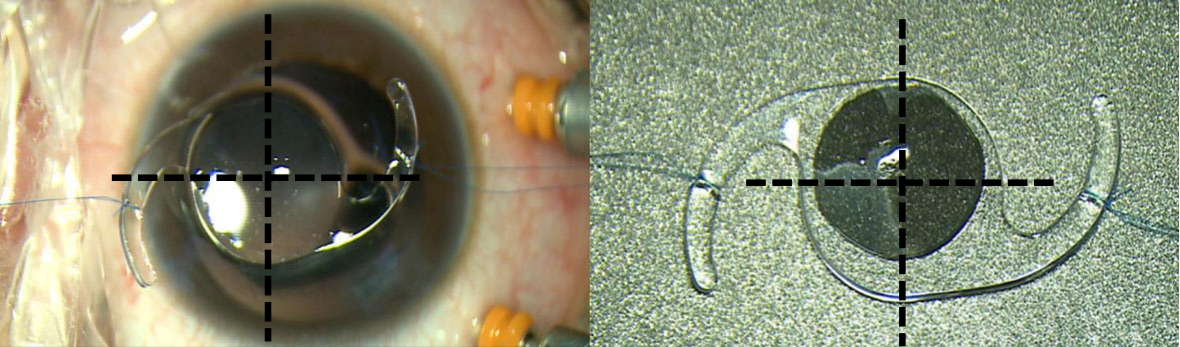
In vitro photos of the 10–0 polypropylene fixation suture knot around the haptic of a one-piece chamber intraocular lens

Many studies have reported that three-piece and one-piece IOL were used as the SFIOL [33, 34]. To be fair, the three-piece lens is more suitable to fix by sutureless scleral tunnel which is similar with Yamane technique [11]. If three-piece IOL were fixed by suture, there are still some questions to be considered. First, the hepatics of three-piece IOL is hard and smooth, which is easy to slip off. Second, If the three-piece IOL enters and exits repeatedly in the injector, it is easy to bend the hepatics or make a crease in optic part of the lens. Third, according to the published literature, suture fixation of one-piece lens can also achieve comparable results with three-piece IOL [35–38]. People usually worry about that one-piece SFIOL will lead to more complications including IOL instability, wide iris-haptic and iris optic contacts, and inflammatory reactions compared with three-piece SFIOL. But from the existing articles and our results, no evidence indicated that there is a significant difference in complications between one-piece and three-piece SFIOL. In fact, similar to previous reports [39–41]. Our long-term results showed that one-piece SFIOL did not happen more complications than that of three-piece SFIOL. On the contrary, we can avoid suture slip off through making a shallow groove on the haptic of one-piece IOL (Fig.  4). If the ligation is too tight, it may be cut the one-piece acrylic haptic. However, it needs to tight with more power. In our technique, only a shallow groove was formed by ligation on the haptics and there was no haptic break during our follow-up. Moreover, we did not find that the acrylic haptic was cut through repeated practice after the ligation was unraveled or removed. In addition, double sutures are more difficult to produce a cutting effect than the single suture.

Our technique aims to improve visual outcomes, reduce the complications, stabilize IOL fixation, minimize ocular surface damage, and shorten operation time. This technique precludes the need for conjunctival and sclera dissection, sclerotomy, sutured wound closure, and knot. Although our patients had a complex variety of preexisting ocular conditions, the preoperative to postoperative BCVA was statistically significant (*P* = 0.01). After a long-term, mean 34 months follow-up, there was no evidence of significant SFIOL decentration or severe complications. We also applied this technique to non-traumatic aphakic eyes without adequate capsule support, and the same results were obtained. These data were not included in this study.

## Conclusions

Our minimally invasive knotless SFIOL technique may be useful for aphakic eyes due to traumatic or other causes without sufficient capsule support and provides good visual outcomes as well as stable IOL fixation. It is simple and fast with fewer complications because of no incision of conjunctiva and sclera.

## Additional file

Additional file 1: Supplemental Digital Content. Demonstrates the surgical techniques in this manuscript. (MPEG 19176 kb)

## Data Availability

The datasets used or analysed during the current study are available from the corresponding author on reasonable request.

## Abbreviations

ACIOL: Anterior chamber intraocular lens
BCVA: Best corrected visual acuity
ERM: Epiretinal membrane formation
IOL: Intraocular lens
IOP: Intraocular pressure
logMAR: log of the minimum angle of resolution
PPV: pars plana vitrectomy
SFIOL: Scleral-fixated intraocular lens
